# Analytical Study of Donor's Milk Bank Macronutrients by Infrared Spectroscopy. Correlations With Clinic-Metabolic Profile of 100 Donors

**DOI:** 10.3389/fpubh.2019.00234

**Published:** 2019-09-12

**Authors:** Stefania Sbrizzi, Pasqua Anna Quitadamo, Domenico Ravidà, Giuseppina Palumbo, Pier Paolo Cristalli, Massimo Pettoello-Mantovani

**Affiliations:** NICU, Casa Sollievo della Sofferenza Hospital, San Giovanni Rotondo, Italy

**Keywords:** donor human milk, human milk bank, breast milk, macronutrients human milk, clinic-metabolic profile of donors, preterm newborns, infrared spectroscopy

## Abstract

For its specific qualitative characteristics human donor milk (DM) is the main alternative to preterm infants nutrition and growing. How several studies suggest child's physical and mental development is influenced by breastfeeding that prevents the necrotizing enterocolitis (NEC), retinopathy of prematurity (ROP), and sepsis common in preterm newborns. Our research was conducted in NICU's Human Milk Bank (HMB) “Allattiamolavita.” Our study was based on macronutrients analysis (MA) of 100 DM samples taken until 10 days since childbirth and analyzed by spectroscopic infrared innovative method (MIRIS). This is a specific method to analyse fat (F), crude proteins (CP), true proteins (TP), carbohydrate (CHO), and total solids (TS). We also analyzed the 100 donors' clinic-metabolic profile by blood tests (PMT). Both data was collected between September 2015 and February 2018. The research was structured in two parts. In the first part we compared PMT with qualitative and quantitative characteristics of MA while in the second one we studied every DM macronutrient finding furthermore possible relations between them. Statistical Package for Social Sciences (SPSS-IBM 24) was used to compare data and reporting coefficient of determination using Spearman's Rho and Kendall's Tau. We also analyzed samples using Kolmogorov–Smirnov test, Student *T*-test, ANOVA, Whitney *U*-test, and chi-square test. Statistically significant correlations were noted between maternal serum proteins and CP—TP of DM. The research showed also significant correlations between azotaemia and TP and an inverse correlation between serum creatinine and CP. No statistically significant correlation was observed between donors' glycaemia and CHO. Mineral concentrations of DM emerged independent of blood minerals (P, Ca, Fe, Na). We also calculated a normal range for individual macronutrient of human transitional milk (TM) that was not established in literature yet. Our experience allowed us to underline that human milk is a privileged site compared to donors' clinical and metabolic disorders. Our analysis showed the major role of the HMB to provide DM to improve clinical status, growing, and neurocognitive short and long term outcome of preterm infants.

## Introduction

Breast milk (BM) is the first-choice food for the preterm newborn: it brings important benefits at gastrointestinal, immunological, nutritional, and cognitive levels (1). When this food is not available or not sufficient, such as in cases of very critical maternal conditions or transient separation by transfer of the newborn, DHM is the most suitable alternative. The main advantages of using BM in the diet of preterm infants are: low incidence of necrotizing enterocolitis (2, 3), reduced incidence of sepsis and other infections (4–7), reduced incidence of bronchopulmonary dysplasia (8), high food tolerance (9), prevention of arterial hypertension and insulin resistance (10, 11). The national guideline on the protection, promotion, and support of breastfeeding states that “breast milk is, where not contraindicated, the most appropriate food for the nutritional needs of premature and hospitalized infants” (12). Breast milk is currently being researched—several new bioactive components have been identified and described, thanks to the advancement of biotechnology (13). It is important to focus on the aspects of its composition in order to optimize the use, especially for the VLBW for whom it is considered a life-saving drug (14, 15).

## Purpose

This study aims to:

- establish ranges of normal values for human transitional milk;
- research possible correlations between the clinical-metabolic situation of mothers and the nutritional values of their milk;
- analyze individual macronutrients of donor human milk (DHM);
- search for possible correlations between the above nutrients;
- study how to positively deal with the caloric-energetic contribution of BM.

## Methods

The Human Milk bank (HMB) “AllattiamolaVita” of the Hospital “Casa Sollievo della Sofferenza” (CSS) in San Giovanni Rotondo is part of NICU. It provides the donated human milk for the feeding of preterm newborns on medical prescription. A database containing all the data useful for the objectives of the research has been created at the beginning of our study, namely:

- identification code referred to units;
- personal data of mothers: date of birth, age at the delivery, date of donation to HMB;
- clinical situation of the pregnant woman;
- date of hospitalization for delivery;
- fill personal forms “Donors' Form”: free donation of milk, explain exclusion criteria;
- infection of HBV, CMV, HIV, HCV, Syphilis, smoke, drugs and tattoos, piercing and surgical operations on last 6 months; (Image 1)
- blood tests of mothers;
- macronutrients of DHM.

The status of the blood tests has been defined according to the reference parameters of the CSS Analysis Laboratory.

Laboratory examinations on undiluted human milk were carried out without additives at the HMB of the same hospital, via Miris-HMA, an infrared spectroscopic analyzer. This method permitted to detect, specifically: fat (F), crude-proteins (CP), carbohydrates (CHO), total solids (TS), true-proteins (TP), and energy (E).

## Participants and Procedures

The analysis of the blood tests of 100 women who have given birth at the CSS Gynecology and obstetrics department are related to glucose, azotemia, creatininemia, total proteins, sodium, phosphorus, calcium, and iron. The macronutrients of 100 samples taken within the 17th post-partum day, were subsequently analyzed by the methodical MIRIS. The study included samples of fresh donated milk and stored refrigerated milk at −20°C. Both analyses were carried out between September 2015 and February 2018. In detail, correlations have been sought between:

- levels of maternal blood glucose and CHO concentration in DHM;
- levels of maternal azotemia and TP in DHM;
- levels of maternal creatininemia and CP in DHM;
- maternal serum proteins and CP-TP proteins of DHM;
- maternal blood trace elements and mineral concentration of DHM;
- maternal iron and macronutrients of DHM.

## Statistical Analysis

The group comparison was made using the statistical software Package for Social Sciences version 24 (SPSS-IBM). The results were expressed as mean ± standard deviation with confidence interval to 95% for continuous variables and as percentages for categorical and discrete variables. The computation of the Spearman Rho coefficients and the Kendall Tau β were performed to assess the degree of correlations between the variables. The Kolmogorov–Smirnov test was used to sample the normality of the data. Finally, the data were analyzed by Student *T*-tests, ANOVA, Whitney *U*-test, and Chi-Quadro test. Statistically significant values were considered with *p* < 0.05.

## Results

### Analysis of the Blood Tests

The data obtained in our study are presented in Tables 1–**5**. The tested blood glucose is 77.29 ± 14.80 mg/dl, range 53.00–151.00 mg/dl, and median 76.00 mg/dl. In the sample analyzed, 30 subjects report a condition of hypoglycemia (30% of the general population), 6 subjects (6%) hyperglycemia, and the remaining 64 (64%) average blood glucose. Serum nitrogen is 18.75 ± 5.14 mg/dl, range 6.00–34.00 mg/dl, and median 18.00 mg/dl. In the general population 18 donors (18% of the sample) report azotemia values below the lower limit and the remaining 82% (82 subjects) report the standard serum values. The creatininemia values of people belonging to the population are 0.55 ± 0.11 mg/dl, with range 0.18–0.94 mg/dl, and median 0.93 mg/dl. In the sample, 50% (50 subjects) of the donors report the creatininemia below the normal range, the remaining 50 (50%) have values compatible with the standard. Serum values of total proteins within the entire population are 6.66 ± 0.44 g/dl, with a range of 5.66–7.52 g/dl, and median 6.70 g/dl. In the sample, 30 donors (30%) show blood concentrations of total proteins below the normal range. The sodium blood concentration is equal to 138.11 ± 2.09 mMol/L, with a range of 132.00 ± 143.00 mMol/L, and median of 138.00 mMol/L. Ninety-two patients (92%) have normal sodium blood values while the remaining 8 (8%) show mild hyponatremia conditions (132 mMol/L). The blood values of phosphorus showed the average of 3.23 ± 0.48 mg/dl, range 2.20–4.60 mg/dl with median 3.20 mg/dl. Six percent (3 donors) of the blood phosphorus test has a ipofosforemia condition, while the remaining 94% (47 donors) has blood phosphorus levels compatible with the standard. Serum calcium of 51 blood samples with an average of 8.77 ± 0.37 mg/dl, range 8.10–9.59 mg/dl, and median 8.80 mg/dl was evaluated. The totality of the population in analysis, 51 subjects, has ordinary calcium values. Finally, in a sub-sample of 80 subjects, the iron was found to be equal to 84.10 ± 42.33 mcg/dl, with range 21.00–221.00 mcg/dl, and median 75.00 mcg/dl. A partial deficiency is reported in 21 individuals (26.25% of the entire sub-sample), 3 individuals (3.75%) have an increase of the iron values (>221 mcg/dl) and the remaining 56 (70.00%) show iron blood values in the standard.

**Table 1:** Descriptive statistics of blood glucose, azotemia, creatitinemia, and total maternal serum proteins and concentrations of carbohydrates, true proteins, crude proteins, and energy of DHM.

	***N***	**Minimum**	**Maximum**	**Middle**	**Standard deviation**
CHO in hypoglycemic	30	1.40	8.50	5.1733	1.90334
CHO in normoglycemic	64	1.40	8.50	5.3953	1.69719
CHO in hyperglycemic	6	5.00	6.80	6.2500	0.66858
Number of valid cases	100				
TP in ipoazotemic	18	0.40	1.90	1.2056	0.37647
TP in normoazotemic	82	0.08	3.60	1.0654	0.60167
Number of valid cases	100				
CP in ipocreatininemic	50	0.50	5.70	1.6300	1.03967
CP in normocreatininemic	50	0.10	4.20	1.3500	0.95538
Number of valid cases	50				
Total proteins low	34	5.66	6.48	6.1665	0.20084
CP in total proteins low	34	0.10	1.80	0.8906	0.49541
TP in total protein low	34	0.10	4.40	1.1824	0.78450
Total proteins normal	66	6.50	7.52	6.9202	0.27871
CP in total proteins normal	66	0.10	5.70	1.6485	1.07033
TP in total proteins normal	66	0.08	3.60	1.1936	0.57994
Number of valid cases	100				
Energy	100	6.00	110.00	57.3100	17.58598
Number of valid cases	100				

### Population Overview and Nutrient Analysis

It was observed that:

- lipids of the 100 samples are 3.26 ± 1.77 g/dl, with range 0.20–9.70 g/dl, and median 2.90 g/dl;
- the full sample CPs are 1.49 ± 1.00 g/dl, with range 0.10–5.70 g/dl, and median 1.30 g/dl;
- CHO have an average value of 5.36 ± 1.72 g/dl, range 1.40–8.50 g/dl, and a median of 6.10 g/dl;
- the proportion of minerals in the BM tested is 10.10 ± 2.86 g/dl, with range 1.20–17.10 g/dl, and median 10.61 g/dl;
- total sample energy is included in a range of 6.00–110.00 g/dl, with an average value of 57.31 ± 17.59 g/dl, and median 56.21 g/dl;
- TP are 1.09 ± 0.57 g/dl, with range 0.8–3.60 g/dl, and median 1.00 g/dl (**Table 5**).

### Blood Glucose Levels and Correlation With CHO Concentration in DHM

The first correlation is between levels of blood glucose and CHO concentration. We analyzed the data of the entire population and the related milk samples, noting that:

➢ 30 subjects have blood glucose values below normal values (70–100 mg/dl):
  ▪ carbohydrate levels in milk equal to 5.17 ± 1.90 g/dl with range 1.40–8.50 g/dl;
  ➢ 6 subjects have blood glucose values above the normal values:
  ▪ carbohydrate levels in milk equal to 6.10 ± 0.34 g/dl with range 6.10–6.80 g/dl;
  ➢ 64 subjects have glucose values in the standard:
  ▪ carbohydrate levels in milk equal to 5.40 ± 1.70 g/dl with range 1.40–8.50 g/dl (Table 1).

The correlation between blood glucose and carbohydrate concentration in DHM was statistically insignificant according to the coefficient of Spearman (*p* = 0.093; Table 2). We compared, moreover, the averages of carbohydrates between hypoglycemic donors and hyperglycemic ones: they were found to be not statistically different from the Student *T*-Test (*p* = 0.131).

**Table 2:** Statistical analysis by Rho Spearman of the correlations between azotemia and true proteins, creatitinemia, and crude proteins, maternal total blood protein, respectively, with crude proteins and true proteins of BM.

			**Azotemia**	**TP**
Rho of Spearman	Azotemia	Correlation coefficient	1.000	−0.259^**^
		Sign. (two tails)	.	0.009
		*N*	100	100
	TP	Correlation coefficient	−0.259^**^	1.000
		Sign. (two tails)	0.009	.
		*N*	100	100
			**Creatininemia**	**CP**
Rho of Spearman	Creatininemia	Correlation coefficient	1.000	−0.246^*^
		Sign. (two tails)	.	0.014
		*N*	100	100
	CP	Correlation coefficient	−0.246^*^	1.000
		Sign. (two tails)	0.014	.
		*N*	100	100
			**Total proteins**	**CP**
Rho of Spearman	Total proteins	Correlation coefficient	1.000	0.241^*^
		Sign. (two tails)	.	0.016
		*N*	100	100
	CP	Correlation coefficient	0.241^*^	1.000
		Sign. (two tails)	0.016	.
		*N*	100	100
			**Total proteins**	**TP**
Rho di Spearman	Total proteins	Correlation coefficient	1	0.232^*^
		Sign. (two tails)		0.020
		*N*	100	100
	TP	Correlation coefficient	0.232^*^	1
		Sign. (two tails)	0.020	
		*N*	100	100

* *The correlation is significant at level 0.05 (two-tailed)*.

** *The correlation is significant at level 0.01 (two-tailed)*.

### Azotemia and Correlation With TP in DHM

On 100 samples analyzed:

➢ 18 donors have azotemia values below normal range (15–38 mg/dl):
  ▪ true protein levels in milk equal to 1.21 ± 0.38 g/dl, with range 0.40–1.90 g/dl;
➢ 72 donors present blood nitrogen values compatible with the normal range:
  ▪ true protein levels in milk equal to 1.07 ± 0.60 g/dl, with range 0.80–3.60 g/dl (Table 1).

We have demonstrated the presence of an inverse correlation between Azotemia and concentration of True proteins by the coefficient of Spearman (*p* = 0.009; Table 2).

### Creatininemia and Correlation With the CP of DHM

The subsequent step is represented by the analysis of the blood creatinine levels and the concentrations of crude proteins. We observed that:

➢ 50 donors have values below the normal range of creatininemia (0.55–1.02 mg/dl):
  ▪ levels of crude protein are equal to 1.63 ± 1.04, with range 0.5–5.70 g/dl;
➢ 50 remaining donors have normal values of Creatininemia:
  ▪ levels of crude protein are equal to 1.35 ± 0.14 g/dl, with range 0.10–4.20 g/dl (Table 1).

The correlation coefficient has shown a reverse condition between creatininemia and crude protein concentrations (*p* = 0.014; Table 2).

### Correlations Between Serum Proteins and Crude-True Proteins

The following step was to look for possible correlations between the total serum proteins and the CP-TP of the BM in analysis. We have therefore observed that:

➢ 30 subjects have total protein values below the standard (6.40–8.20 g/dl):
  ▪ the concentrations of crude proteins in DHM are equal to 0.89 ± 0.50 g/dl with range 0.10–1.80 g/dl;
  ▪ the concentrations of true proteins are instead of 1.18 ± 0.78 g/dl with range 0.10–4.40 g/dl;
➢ 70 subjects have total protein blood values in the standard, moreover:
  ▪ the concentrations of crude proteins are equal to 1.65 ± 0.28 g/dl, with range 0.10–5.70 g/dl;
  ▪ the concentrations of true proteins are equal to 1.19 ± 0.58 g/dl with range 0.08–3.60 g/dl (Table 1).

We therefore observed that there are statistically significant correlations between the crude protein concentrations and the total blood proteins, and between the latter and the true proteins, both represented by the correlation coefficient (respectively, *p* = 0.016 and *p* = 0.020; Table 2). It was thus demonstrated by the Student *T*-test that the two averages of the crude protein concentrations (with total blood proteins below the standard and in the norm) are statistically different from each other (respectively, with *p* = 0.006 with test of Leneve and *p* = 0.024 with *T*-Test for the comparator of the averages; Table 3). The same statistically significant correlation was, finally, demonstrated with the true proteins by means of the Student *T*-Test (with respectively, *p* = 0.006 with test of Leneve and *p* = 0.007 with tests of equality of the averages; Table 3).

**Table 3:** Student *T*-Test for independent samples between the concentrations of crude and true proteins of DHM in the two levels of total maternal blood proteins.

		**Test of Levene**		**Test** ***t*** **for the equality of the averages**						
		***F***	**Sign**.	***t* independent**	**gl**	**Sign. (two tails)**	**Difference middle**	**Difference error standard**	**Range of confidence of the 95% difference**	
									**Inferior**	**Superior**
Total proteins	Var. equal alleged	7.783	0.006	−12.983	98	0.000	−0.75890	0.05846	−0.87491	−0.64290
	Var. equal not alleged			−15.391	83.087	0.000	−0.75890	0.04931	−0.85698	−0.66083
CP	Var. equal alleged	1.235	0.269	−2.102	98	0.038	−0.45238	0.21525	−0.87954	−0.02522
	Var. equal not alleged			−2.310	68.97	0.024	−0.45238	0.19586	−0.84312	−0.06164
Total proteins	Var. equal alleged	7.783	0.006	−12.983	98	0.000	−0.75890	0.05846	−0.87491	−0.64290
	Var. equal not alleged			−15.391	83.087	0.000	−0.75890	0.04931	−0.85698	−0.66083
TP	Var. equal alleged	0.052	0.820	−2.579	98	0.011	−0.31133	0.12071	−0.55088	−0.07179
	Var. equal not alleged			−2.777	65.525	0.007	−0.31133	0.11211	−0.53521	−0.08746

### Correlations Between Blood Trace Elements and the Concentration of Minerals

It also sought to demonstrate the possible correlations between calcium, sodium, blood phosphorus, and the concentration of minerals. With regard to blood calcium concentrations we analyzed a sub-sample of 51 subjects and the corresponding samples of BM. It was therefore observed that there are no statistically significant correlations between the blood calcium and the concentrations of the minerals in the milk (*p* = 0.116). The totality of the sample has been analyzed as regards the possible correlations between blood sodium and the concentration of minerals. It has therefore been shown that there are no statistically significant correlations between these two independent variables (*p* = 0.327). Finally, the possible correlation between phosphorus levels and the concentration of minerals in BM samples was sought by analyzing a sub-sample of 50 individuals within the general population with their corresponding milk.

### Correlations Between Iron and Nutrients of BM

We also sought the possible correlations of iron with each nutrient of DHM observing iron serum levels of 84.10 ± 4.73 mcg/dl, range 21.00–221.00 mcg/dl, and median 75.00 mcg/dl. No statistically significant correlations between the iron and each nutrient of DHM have been highlighted.

### Descriptive Analysis of the Components of Transitional DHM

In this part of the study we focused on the macronutrient composition of DHM samples. We established a range of concentrations for each nutrient and the possible correlations between the variables in question, i.e., fat, carbohydrates, crude proteins, true proteins, total solids, and energy of DHM have been changed. Considering the caloric intake of transitional DHM, it was observed that:

- energy levels are represented by 53.31 ± 17.59 g/dl, with range 6.00–110.00 g/dl, and with median 56.21 g/dl;
- statistically significant correlations between the energy and the other nutrients of the 100 samples can be pointed out (Table 4).

**Table 4:** Statistically significant correlations between energy and other nutrients of BM.

			**Energy**	**Fat**	**CP**	**CHO**	**Total solids**	**TP**
Rho of Spearman	Energy	Correlation coefficient	1.000	0.797^**^	0.428^**^	0.229^*^	0.872^**^	0.330^**^
		Sign.	.	0.000	0.000	0.022	0.000	0.001
		*N*	100	100	100	100	100	100
	Fat	Correlation coefficient	0.797^**^	1.000	0.235^*^	−0.099	0.533^**^	0.088
		Sign.	0.000	.	0.018	0.326	0.000	0.382
		*N*	100	100	100	100	100	100
	CP	Correlation coefficient	0.428^**^	0.235^*^	1.000	0.108	0.531^**^	0.887^**^
		Sign.	0.000	0.018	.	0.283	0.000	0.000
		*N*	100	100	100	100	100	100
	CHO	Correlation coefficient	0.229^*^	−0.099	0.108	1.000	0.494^**^	0.234^*^
		Sign.	0.022	0.326	0.283	.	0.000	0.019
		*N*	100	100	100	100	100	100
	Total solids	Correlation coefficient	0.872^**^	0.533^**^	0.531^**^	0.494^**^	1.000	0.489^**^
		Sign.	0.000	0.000	0.000	0.000	.	0.000
		*N*	100	100	100	100	100	100
	TP	Correlation coefficient	0.330^**^	0.088	0.887^**^	0.234^*^	0.489^**^	1.000
		Sign.	0.001	0.382	0.000	0.019	0.000	.
		*N*	100	100	100	100	100	100

** *The correlation is significant at level 0.01 (two-tailed)*.

* *The correlation is significant at level 0.05 (two-tailed)*.

## Discussion

Breast Milk is a complex and dynamic fluid comprising numerous bioactive factors and different cellular populations making it the natural food par excellence, optimal for the newborn and for the preterm infant (16–19). One of the strengths of our analysis consists in the infrared spectroscopy because it does not foresee neither coloration nor dilution of the sample with a good preservation of the properties of the nutrients promoting the collection and the analysis of more accurate and reliable data (20).

The study is divided into two main phases. In the first phase, the possible influence of the clinical-metabolic structure of the mothers on the qualitative and quantitative characteristics of DHM nutrients was sought. The second phase was based, instead, on the study of the macronutrients of human milk and their possible correlations. The first figure that emerges from our results is the non-correlation between donor blood glucose and the glucose profile of the corresponding milk samples. In fact, there is no statistically significant correlation between the blood glucose itself and the carbohydrates (*p* = 0.093).

Another datum in favor of this thesis is the non-statistically significant difference of the average concentrations of the milk carbohydrates grouped by conditions of hypo/normo/hyperglycemia (*p* = 0.131). At this point we can say that it would be interesting to increase the number of the sample in analysis because, despite being not statistically significant, the increase in blood glucose showed a slight increase in the average concentration of carbohydrates and a gradual reduction in the dispersion of relative values (s.d.).

Our sample presented a close correlation between azotemia and true proteins of DHM (*p* = 0.009). In particular, it was observed that between these two variables there is a reverse relationship represented by the reduction of true proteins in response to the increase of azotemia. As shown in literature, the nitrogen balance, in the assessment of the nutritional status, is calculated as the difference between the intake and the elimination of the nitrogen itself. This balance is positive during pregnancy and breastfeeding but becomes negative in the case of insufficient protein and energy intake and when there is an imbalance between essential and non-essential amino acids (21). It is therefore important not to increase the intake of nitrogen in order to ensure a correct concentration of true proteins in BM.

The data analyzed later were related to creatininemia. A reverse correlation was observed regarding the crude proteins of the corresponding samples of DHM, statistically significant (*p* = 0.014).

We can therefore say that it would be desirable to maintain levels of creatininemia on the lower threshold of the normal limit as this would result in an increase of the concentration of crude proteins within BM. We also remember, as found in the literature, that the latter are responsible for the actual protein content necessary for the correct nourishment of the newborn (21). Another blood test taken into account regarded the total serum proteins. We have researched how these could affect the concentration of macronutrients of human milk. We therefore found a correlation between the total serum proteins (SP) and the protein categories of the milk. Both crude and true proteins are proportional to the value of total blood proteins (*p* = 0.016 and *p* = 0.020, respectively). In this regard, observing that the sample showed 34 values of total proteins lower than the normal range, we divided the general sample into two subsamples relative to the value of the SP calculating the average concentrations of the corresponding rates of BM.

We also found average concentrations between the two statistically different subsamples to confirm the above (*p* = 0.006 and *p* = 0.024). The monitoring of total serum protein values during pregnancy would be useful for a correct protein intake in BM.

We continued our study by analyzing sodium, phosphorus, calcium, and blood iron. We expected to find a close correlation between these and the macronutrients of milk. In particular, a regulation of BM minerals was expected through blood calcium.

These expectations were refuted because all the parameters taken into account, were not statistically correlated with the macronutrients of human milk.

These latest evidences associated with blood glucose results demonstrate that milk is certainly a privileged site because its nutritional characteristics cannot change on the basis of clinical-metabolic alterations.

After the first part of the study, we focused exclusively on milk and its components. Assessed the number of the sample, once the normality of the data has been obtained by means of the Kolmogorov-Smimoy test, we have set ourselves to bring back a range of normal values of the human transitional milk (Table 5).

**Table 5:** Prospectus of normal values of macronutrients of human transitional milk.

Fat	1.49–5.03 g/dl
Crude protein	0.49–2.49 g/dl
Carbohydrate	3.64–7.08 g/dl
Total solids	7.24–12.96 g/dl
Energy	39.72–74.90 g/dl
True protein	0.52–1.66 g/dl

Then we analyzed the possible correlations between the macronutrients of human milk, discussing in particular on the caloric aspect. We can say that all macronutrients play a fundamental role in the caloric-energetic contribution of breast milk, showing statistically significant positive correlation coefficients.

Significant importance in the increase of caloric intake is given by fats and true and crude proteins, according to what is known in the literature (*p* < 0.001, *p* = 0.001, and *p* < 0.001, respectively) (22).

Finally, we found that carbohydrates contribute to caloric intake with a lower significance (*p* = 0.020).

## Conclusion

This study has given us the opportunity to understand and demonstrate how much BM is a privileged site in relation to certain alterations of maternal biochemical profile. Nitrogen and creatinine are implicated in this process, having observed a reverse proportionality with the increase of true and crude proteins and, respectively, the reduction of the azotemia and the serum creatitinemia (*p* = 0.009 and *p* = 0.014; Table 2). Moreover, by observing a directly proportional correlation between the total blood proteins and the overall protein structure of the BM (*p* = 0.016 and *p* = 0.020) during pregnancy and breastfeeding a more careful monitoring of these parameters may be useful (Table 2). It would be desirable to continue these studies by increasing the number of the sample and including metabolic growth and clinical parameters of the newborn. Finally, we want to emphasize the role of the HMB which allows preterm infants to benefit from DHM. This proves to be an irreplaceable resource, as widely promoted during the course of this study, for the improvement of the clinical conditions, for the growth and the short and long term outcomes of our newborns (23–29).

## Author Contributions

SS: introduction, analytical study, results, discussion, and conclusion. PQ: participants and procedures, conclusion. DR: statistical analysis, results, discussion, and conclusion. MP-M: introduction. GP: participants and procedures. PC: introduction.

### Conflict of Interest Statement

The authors declare that the research was conducted in the absence of any commercial or financial relationships that could be construed as a potential conflict of interest.

## Supplementary Material

Supplementary Image 1 Form filled by all donors' that explain exclusion criteria and free milk donation.

## Abbreviations

BM: Breast Milk
CSS: Casa Sollievo della Sofferenza
DHM: Donor Human Milk
HMB: Human Milk Bank
VLBW: birth weight < 1,500 g.
