# Supplementary figures and images for: Analytical Study of Donor's Milk Bank Macronutrients by Infrared Spectroscopy. Correlations With Clinic-Metabolic Profile of 100 Donors
Source: Front Public Health. 2019 Sep 12;7:234. doi: 10.3389/fpubh.2019.00234 (PMC6752055; doi:10.3389/fpubh.2019.00234)

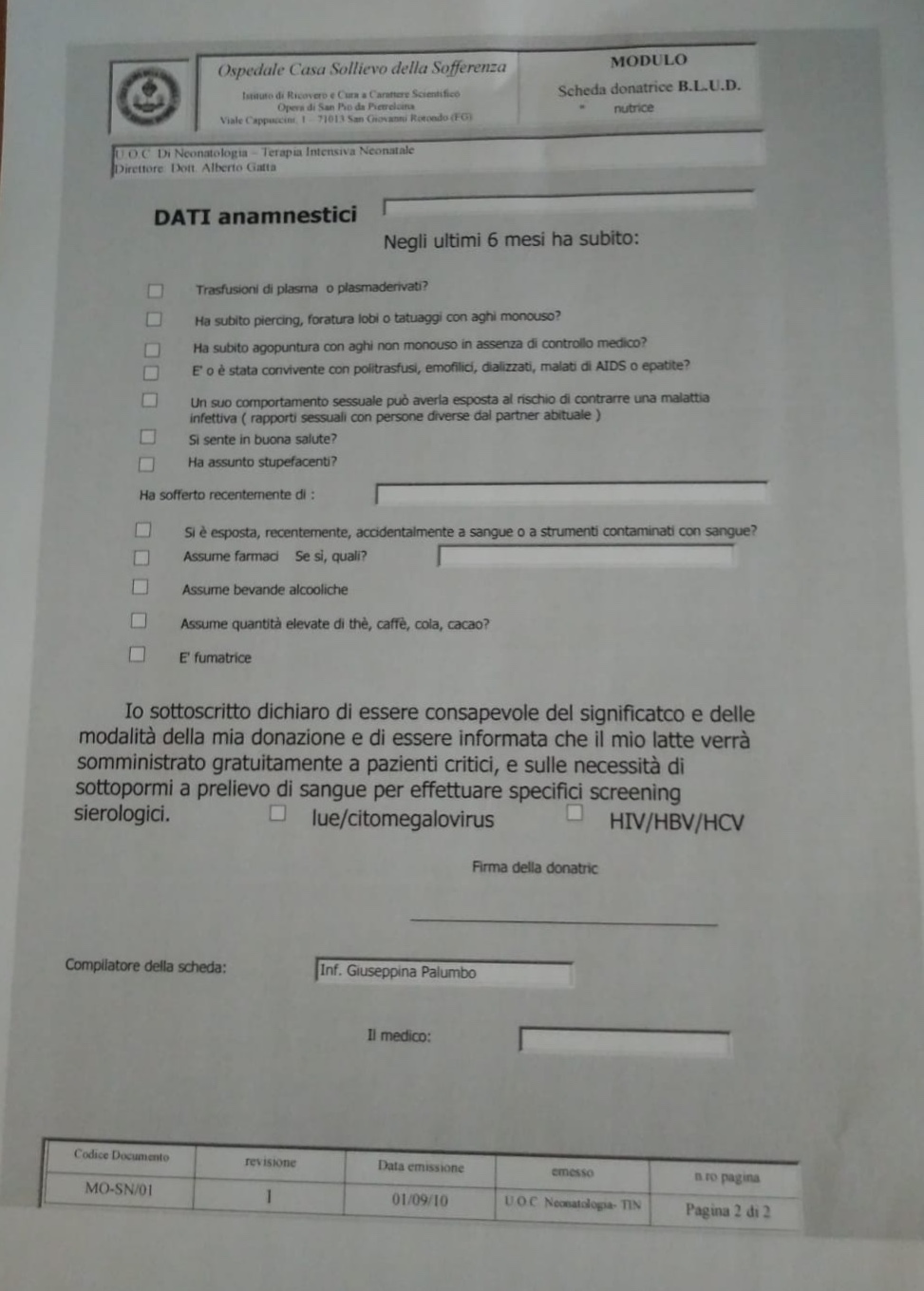

Supplement: Supplementary Image 1 — Form filled by all donors' that explain exclusion criteria and free milk donation. [file Image_1.JPEG]
